# Emergence of High-Level Gentamicin Resistance in *Streptococcus agalactiae* Hypervirulent Serotype IV ST1010 (CC452) Strains by Acquisition of a Novel Integrative and Conjugative Element

**DOI:** 10.3390/antibiotics13060491

**Published:** 2024-05-26

**Authors:** Roberta Creti, Monica Imperi, Uzma Basit Khan, Alberto Berardi, Simona Recchia, Giovanna Alfarone, Giovanni Gherardi

**Affiliations:** 1 Dipartimento di Malattie Infettive, Reparto di Antibiotico-Resistenza e Patogeni Speciali, Istituto Superiore di Sanità, 00161 Rome, Italy; monica.imperi@iss.it (M.I.); simona.recchia@iss.it (S.R.); giovanna.alfarone@iss.it (G.A.); g.gherardi@unicampus.it (G.G.); 2 Parasites and Microbes Programme, The Wellcome Sanger Institute, Wellcome Genome Campus, Hinxton, Cambridge CB10 1SA, UK; uk1@sanger.ac.uk; 3 Neonatal Intensive Care Unit, Department of Medical and Surgical Sciences of Mothers, Children and Adults, University of Modena and Reggio Emilia, 41125 Modena, Italy; alberto.berardi@unimore.it; 4 Unità di Ricerca di Scienze Batteriologiche Applicate, Facoltà Dipartimentale di Medicina e Chirurgia, Università Campus Bio-Medico, 00128 Rome, Italy

**Keywords:** *Streptococcus agalactiae*, GBS, high-level gentamicin resistance (HLGR), ST1010, serotype IV, CC452, mobile genetic element

## Abstract

*Streptococcus agalactiae* (group B streptococci, GBS) is responsible for severe infections in both neonates and adults. Currently, empiric antimicrobial therapy for sepsis and meningitis is the combined use of penicillin and gentamicin due to the enhanced bactericidal activity. However, high-level gentamicin resistance (HLGR) abrogates the synergism. The rate of HLGR was investigated within a dataset of 433 GBS strains collected from cases of invasive disease in both adults and neonates as well as from pregnant carriers. GBS isolates (n = 20, 4.6%) presented with HLGR (gentamicin MIC breakpoint >1024 mg/L) that was differently diffused between strains from adults or neonates (5.2% vs. 2.8%). Notably, 70% of HLGR GBS strains (14 isolates) were serotype IV. Serotype IV HLGR-GBS isolates were susceptible to all antibiotics tested, exhibited the alpha-C/HvgA/PI-2b virulence string, and belonged to sequence type 1010 (clonal complex (CC) 452). The mobile element that harbored the HLGR *aac*(6′)-*aph*(2)″ gene is a novel integrative and conjugative element (ICE) about 45 kb long, derived from GBS 515 ICE tRNA^*Lys*^. The clonal expansion of this HLGR hypervirulent serotype IV GBS CC452 sublineage may pose a threat to the management of infections caused by this strain type.

## 1. Introduction

*Streptococcus agalactiae*, also named group B streptococcus (GBS), is a major bacterial cause of neonatal infections causing early-onset disease (from birth to six days of age; EOD) and late-onset disease (7–89 days; LOD), such as sepsis and meningitis [1,2]. Intrapartum antibiotic prophylaxis (IAP) with beta lactam antibiotics in women colonized with GBS at the vaginal/rectal site has dramatically reduced the burden of EOD [3]. GBS has also been associated with increased rates of invasive and non-invasive infections in adults, especially in elderly patients or in patients with underlying conditions [4].

Ten structurally and antigenically distinct capsular GBS polysaccharides (types Ia, Ib, and II to IX) have been described [5]. All serotypes can cause invasive infections; however, six serotypes (Ia, Ib, II, III, IV, and V) account for most of the disease in neonates, infants and adults [1,6].

Antimicrobial resistance in GBS has been reported mainly to tetracycline, erythromycin, and clindamycin, with an increased rate of erythromycin and clindamycin resistance described worldwide [4,7]. Indeed, GBS isolates with reduced or non-susceptibility to β-lactams have also been occasionally reported from invasive and non-invasive infections, associated with mutations in the *pbp2x* gene [8,9,10,11,12]. Penicillin-allergic patients are often treated with clindamycin or vancomycin [13]. Multidrug resistance in GBS is increasingly reported, mainly due to the emergence of a sublineage of clonal complex 17 (CC17) [14]. Emerging antimicrobial resistance, the need for more feasible prevention strategies for low-income settings, and the potential for primary prevention among high-risk adults, have encouraged studies into GBS vaccine development [1,4]. A hexavalent (Ia, Ib, II, III, IV, and V) conjugate vaccine is currently in clinical trials [15,16]. A protein vaccine, constituted by GBS surface adhesins, is also under development [17].

Currently, the empiric antimicrobial therapy for sepsis and meningitis is the combined use of penicillin (or ampicillin) and gentamicin [18]. The bactericidal synergism is ineffective in the case of high-level gentamicin resistance (HLGR), due to acquired aminoglycoside-modifying enzymes. GBS is intrinsically resistant to low-level gentamicin, and surveillance screening schemes usually do not include gentamicin or other aminoglycoside susceptibility testing because no recommendations or breakpoints for testing HLGR are present in the guidelines issued by the Clinical and Laboratory Standards Institute [19] or the European Committee on Antimicrobial Susceptibility Testing (http://www.eucast.org, accessed on 6 May 2024). The prevalence of HLGR in GBS is, therefore, unknown.

Recently, we retrospectively analyzed GBS isolated from different sources over a 2-year period (2019–2021) and found that 11 out of 89 GBS strains (12.3%) exhibited a gentamicin MIC breakpoint ≥ 1000 mg/L [20]. These results were confirmed by verifying the presence of an intact *aac*(6′)-*Ie*-*aph*(2″)-*Ia* gene [20]. Then, HLGR among GBS may emerge as a potential problem and threaten an effective therapy.

Many surface proteins contribute to GBS virulence and are used for virulence profiling, such as the alpha-like protein (Alp) family (Alpha-C, Epsilon, Rib, Alp2/3, Alp4), pili structures (different assemblies of pilus pathogenic islands 1, 2a, 2b), and the hypervirulent adhesin HvgA, responsible for meningeal tropism [21,22,23,24].

The aim of this study was to estimate the frequency of high-level gentamicin resistance as well as susceptibility to other antibiotics in a larger collection of GBS strains isolated both from invasive infections in adults, newborns, and infants, and from colonized pregnant women. HLGR GBS isolates were further analyzed both phenotypically and genotypically to evaluate their serotype, pili content, surface protein genes, hypervirulent adhesin *hvgA* gene.

This investigation revealed the emergence of a serotype IV lineage in which HLGR is contained in a new genetic element.

## 2. Results

### 2.1. Antibiotic Susceptibility

Twenty GBS strains (4.6%) presented HLGR with a gentamicin MIC breakpoint > 1024 mg/L. The resistance was more diffused among adult than neonatal strains (adult invasive strains: 6 HLGR isolates (5.4%); pregnant carrier women: 11 HGLR isolates (5.1%); neonates: 3 HLGR isolates (2.9%)).

Notably, fourteen out of twenty HLGR GBS strains (70%) were serotype IV (four adult invasive isolates, one neonatal invasive isolate, and nine from carriage). The remaining HLGR-GBS were serotype V (three isolates, one from adult disease and two from carriage), serotype III (two neonatal invasive isolates), and serotype Ib (one adult invasive isolate) as shown in Table 1.

A panel of nine antibiotic sensitivity tests revealed that all 20 HLGR isolates were susceptible to benzylpenicillin, vancomycin, teicoplanin, chloramphenicol, and linezolid. The serotype IV HLGR-GBS isolates also demonstrated susceptibility to the remaining antibiotics tested; the other non-serotype IV HLGR-GBS strains exhibited multidrug resistance (MDR). In particular, serotype V HGLR-GBS isolates were resistant to both levofloxacin and tetracycline, with two of them also showing resistance to erythromycin and clindamycin. The only serotype Ib GBS strain was resistant to erythromycin, clindamycin, and tetracycline. Additionally, the two serotype III strains showed resistance to tetracycline.

Tetracycline resistance was mediated by the *tet*(M) genetic determinant in all resistant strains except in serotype Ib, which carried the *tet*(O) gene. Macrolide resistance was conferred by the *erm*(B) gene in all three strains that exhibited it. Notably, in the Ib serotype, the *erm*(B) gene was constitutively expressed, whereas in serotype V, it was inducible.

### 2.2. Virulence Factors and Clonality

PCR screening on the presence of GBS *alpha-C* (*bca*) and alpha-like (*epsilon*, *rib*, *alp2*/*alp3*) surface protein genes, of the pili islands PI-1, PI-2a, and PI-2b, and of the presence of *hvgA* gene in HLGR GBS isolates is reported in Table 2.

Each serotype exhibited distinct surface protein antigens and pilus island gene combinations. Specifically, serotype IV isolates were characterized by the presence of *alpha-C* and *PI-2b* genes; serotype V was associated with the combination of *PI-1 + PI2a* and *alp1* genes; serotype III strains exclusively harbored *rib* and *PI-1 + 2b* genes; serotype Ib presented *PI-2a* and *alpha-C* genes. Additionally, the *hvgA* gene was detected in all HLGR serotype IV and III isolates.

All 14 serotype IV isolates constituted a single CC, featuring sequence type (ST) 1010, which falls under CC452. HLGR isolates of serotype V, III, and Ib belonged to CC19 (ST19), CC17 (ST17), and CC12 (ST12), respectively (Table 2).

### 2.3. Transposon Structure of HLGR GBS Strains and New ICE

All HLGR isolates were further characterized to investigate the transposon structure carrying the gentamicin resistance gene by PCR mapping. The PCR mapping results and primers used are shown in Table 3 and Table 4, respectively.

Only the serotype Ib HLGR-GBS strain exhibited the presence of a Tn3706 element, which is a 4.5 kb fragment containing the *aac*(6′)-*Ie-aph*(2″)-*Ia* gene flanked by the IS256 insertion sequences. This element bears resemblance to Tn4001 and Tn5281, which have been identified in staphylococci and enterococci, respectively [27,28]. Tn3706 transposon has been previously described in the GBS B128 strain [29] and the Japanese serotype III, ST464 (CC23) MRY08-1422 GBS genome [12].

**Figure 1:**
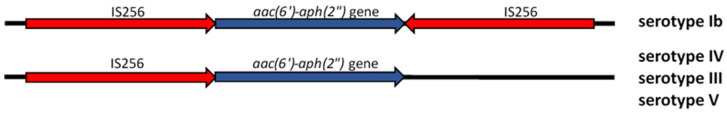
Schematic representations of the different transposon structures found in our HLGR-GBS strains.

All other HLGR-GBS isolates presented the same structure, characterized by a truncated transposon of 3262 bp consisting of an entire IS256 insertion sequence only, located upstream of the gentamicin resistance gene (Figure 1). This truncated form of Tn3706 has also been described in the Japanese serotype III, ST335 (CC19) HU-GB5823 genome [30].

**Figure 2:**
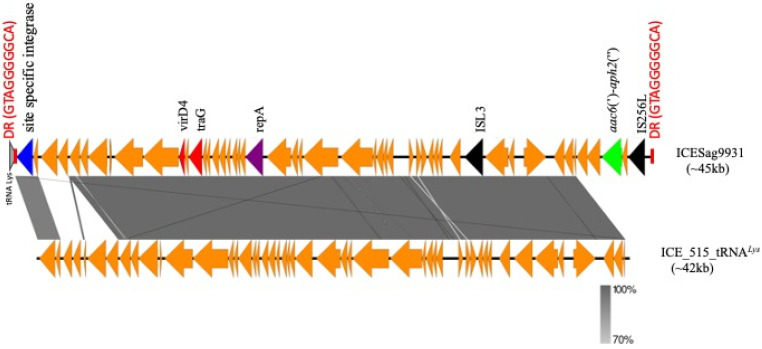
Genomic structure of ICESag9931 and its comparison with ICE*_515_tRNA^Lys^*. The novel ICESag9931 identified in serotype IV ST1010 isolate 9931 in this study was compared for nucleotide similarity with a previously identified ICE*_515_tRNA^Lys^* located at positions 1836154–1878284 in the NZ_CP051004 genome using EasyFig v2.2. In ICESag9931, the *aac*(6′)-*aph*(2″) gene (highlighted in green) with the upstream IS256 element is located at one end of the ICE element.

We focused on delineating the structure of the mobile genetic element containing the truncated Tn3706 transposon of the serotype IV clonal cluster. Whole-genome sequence analysis indicated that this is a ~45 kbp integrative and conjugative element (ICE) that was named ICESag9931, never reported so far (Figure 2).

**Figure 3:**
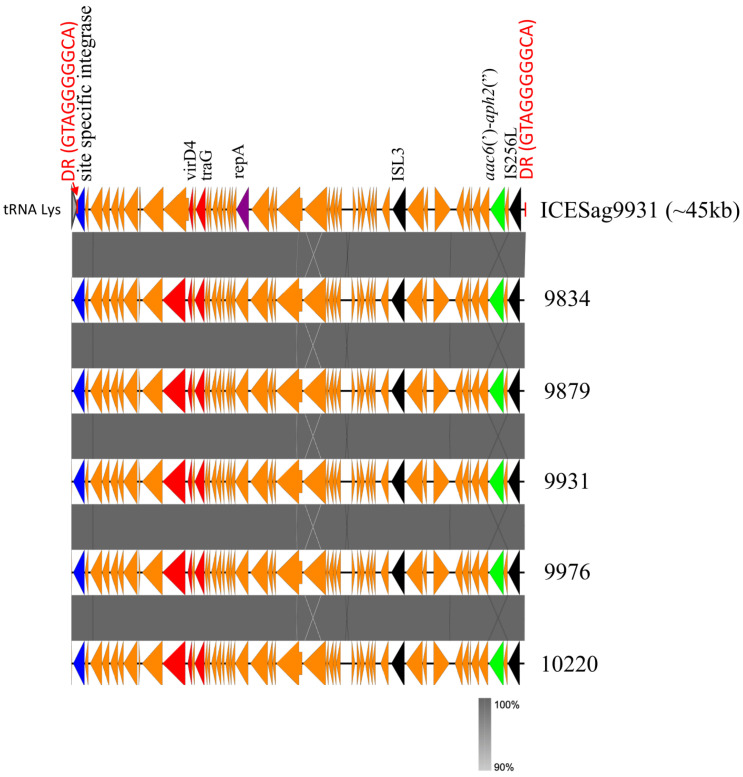
Comparison of ICESag9931 with five serotype IV (9834, 9879, 9931, 9976, and 10220) ST1010 isolates from this study. BLASTn analysis was conducted using Easyfig v2.2.2. Various colors denote significant features, with red vertical lines representing 10 bp direct repeats, dark blue indicating site-specific integrase, red representing conjugal proteins, purple denoting replication initiator protein, black indicating insertion sequences, and green representing antibiotic resistance genes. Grey bars between sequences indicate areas with BLAST hits. The genomic size of ICESag9931 is approximately 45 kbp.

ICESag9931 is the result of the insertion of the truncated Tn3706 transposon on an ICE already described in the GBS 515 strain (ICE*_515_tRNA^Lys^*) that carries different putative virulence genes including one encoding a putative new CAMP factor [31]. A comparative analysis between ICESag9931 and ICE*_515_tRNA^Lys^* revealed an 83% query coverage with 99% nucleotide identity, highlighting their considerable similarity (Figure 2). Additionally, we examined the prevalence of ICESag9931 across the available whole-genome sequences (WGS) of five additional serotype IV isolates, namely, (9834, 9879, 9931, 9976, and 10220). Our investigation unveiled the presence of the *aac*(6′)-*aph*(2″) gene carried by ICESag9931 in all these isolates, demonstrating substantial nucleotide homogeneity with a median of 99.7% (Figure 3).

## 3. Discussion

Antimicrobial resistance represents a primary threat to public health globally. To fight this phenomenon, activities combining antimicrobial stewardship interventions with diagnostic stewardship are mandatory, mainly in hospital settings, to promote the optimal use of antibiotics [32]. Future antibacterial research and development aim to prioritize the discovery of new classes of antibiotics as well as alternative strategies with feasible pathways for market approval and clinical use [33].

Clonal expansion of a GBS lineage that is resistant to high levels of an antibiotic that is not routinely used for GBS infections such as gentamicin adds new concerns in the management and containment measures of the MDR issue.

The mechanism of HLGR resistance in GBS is mediated by the chromosomally integrated transposon Tn3706 (a derivative of enterococcal Tn5281 or staphylococcal Tn4001) or plasmid pIP501 [12,14,29,30,34,35,36,37]. Nevertheless, the HLGR rate in GBS is rarely reported, although probably underestimated due to the absence of international guidelines on HLGR susceptibility testing for GBS. A survey of 6340 isolates recovered during the years 2015–2017 through the population-based Active Bacterial Core surveillance (ABCs) program in the United States demonstrated that the *aac*(6′)-*aph*(2″) gene was present in only 0.27% of GBS strains [22]. Similarly, among 1128 GBS isolates responsible for cases of diseases or colonization and of different geographical origin, only two (0.17%) strains with HLGR were identified [14].

GBS clinical isolates of serotype III, collected at Korea University Hospital (Seoul, Republic of Korea)—not expressing srr1 or srr2 proteins, which are important in bacterial infection—were genotyped. Overall, 11 isolates, all of which belonged to ST19, exhibited higher resistance to gentamicin, kanamycin, and tobramycin (MIC values of 512 mg/mL). One representative isolate analyzed by whole-genome sequencing revealed the presence of four large novel cluster sites. Specifically, site 4 in the srr1 gene locus was replaced by an *lsa*(*E*)*-lnu*(*B*)*-aadK-aac-aph-aadE*-carrying multidrug-resistant gene cluster flanked by two IS1216 transposases with 99% homology to the enterococcal plasmid pKUB3007-1 [37].

A systematic investigation of antimicrobial resistance genomic content to characterize mobile genetic elements was performed in 193 invasive and non-invasive GBS isolates collected from infections of UK adult patients during the years 2014 and 2015. The gentamicin-resistant *aac*(6′)-*aph*(2″) gene was detected in only one CC19 serotype V isolate, carried by the novel ICESag139 [38]. No barriers to ICE transfer between strains exist, also from different hosts, making the acquisition of these resistance traits a threat for human health [39].

The clinical significance of the emergence of HLGR in GBS is not clear either. High-level aminoglycoside resistance abrogates the enhanced bactericidal activity of gentamicin in combination with ampicillin, which is the initial therapy for neonatal sepsis and meningitis [18]. The combination of systemic penicillin plus local gentamicin has also been suggested as a potential application in orthopedic device-associated biofilm GBS infections [40]. We recently recommended that all clinical isolates of GBS should be tested for susceptibility to gentamicin, considering a MIC value ≥ 512 mg/L as an indication for the identification of HLGR GBS, with an alert for gentamicin MIC values between 128 mg/L and 512 mg/L [20].

In this study, a total of 20 HLGR isolates were identified in a dataset of 433 GBS isolates from adult invasive disease, neonatal invasive disease, and from carriage, with a rate of 4.6%. HLGR GBS were more diffused among adults than neonates (5.3% vs. 2.9%). All HLGR isolates were *aac*(6′)-*aph*(2″) gene-positive and the majority were serotype IV (70% of all HLGR GBS isolates). All 14 serotype IV HLGR-GBS isolates were susceptible to all antibiotics tested while multidrug resistance was found in the other HLGR-GBS of serotypes V, III, and Ib.

All serotype IV HLGR-GBS isolates showed the virulence string alpha-C/HvgA/PI-2b. By MLST and WGS, they represented a single lineage (ST1010, a single-locus variant of ST452), an emergent hypervirulent serotype IV clone [41]. The ST452 serotype IV lineage originated from the recombination of GBS ST24 (Ia, CC23) with ST291 (serotype IV, CC17) [41], acquiring a unique combination of virulence factors derived from the parent STs as the *alpha-C* protein gene from ST24, pili island 2b gene only, and *hvgA* gene from ST291. Interestingly, none of the ST452 strains reported so far presented any known antibiotic resistance genes. So, the gentamicin resistance was specifically acquired by ST1010.

The virulence string and clonal lineage of the other HLGR-GBS serotypes identified in this study are in line with findings from previous studies, confirming the already observed specific association between serotype, pili, alpha-like proteins, and clonal complexes [41,42,43,44,45,46,47].

The genetic element carrying the *aac*(6′)-*aph*(2″) gene was identical in all our HLGR serotype IV isolates and was part of a new integrative and conjugative element, named ICESag9931. Indeed, ICESag9931 is identical to an already described ICE identified in the serotype Ia GBS 515 strain, except for the insertion at one side of the truncated Tn3706 element that comprises the HLGR gene.

The increased prevalence of serotype IV, mainly in adult GBS, is an emerging data [48]. In the US, a notable emergence of and increase in serotype IV among non-pregnant adults over time was reported [4,22]. Similarly, in the present study, the prevalence of serotype IV was found to increase in Italy as well [49,50,51,52], mainly due to the clonal expansion of the HLGR ST1010 lineage.

A PubMLST search for GBS ST1010 (26 April 2024) retrieved three strains that were isolated in the US in the year 2016 and seventeen from carriers from the Dominican Republic in the year 2021. All are reported as serotype IV GBS strains. An additional search in Monocle Data Viewer (https://data-viewer.monocle.sanger.ac.uk/project/juno, accessed on 26 April 2024) reported three serotype IV GBS ST1010 strains isolated from vaginal carriage in the Netherlands in the year 2019 and five serotype GBS ST1010 strains from the US, detected in the year 2017. Of notable importance, all GBS strains from the Netherlands and only one from the US are reported as HLGR.

These findings indicate that HLGR GBS serotype IV/ST1010/CC452 may further spread and disseminate, representing a potential threat for the treatment and management of invasive GBS infections.

The clonal expansion of the HLGR serotype IV/CC452 hypervirulent GBS lineage should be monitored over time and HLGR rates among GBS should be included in surveillance screening programs.

## 4. Materials and Methods

### 4.1. GBS Strains Dataset

A total of 433 GBS strains from invasive GBS disease (112 isolates from adults and 104 isolates from newborns and infants up to three months of life) and from pregnant carrier women (217 isolates) were analyzed. Adult and neonatal invasive GBS clinical isolates were collected by the national passive surveillance coordinated by ISS-NRL (years 2016–2020). Isolates from carriage (women antenatal screening) come from the PREPARE study (years 2021–2021). PREPARE is the acronym for the project “PREvention of invasive Group B Streptococcus disease in young infants: a PAthway for the evaluation & licensuRE of an investigational maternal GBS vaccine (PREPARE GBS)”.

Surveillance studies involving only the collection of bacterial samples (not human biological material) are exempt from the authorization of the local/national ethics committee. The PREPARE study received ethical approval from the competent authority (n. 1079/2019).

### 4.2. Bacterial Isolates Typing

Bacterial strains were plated in parallel on defibrinated sheep blood agar plates (Liofilchem, Roseto degli Abbruzzi, Italy) and chromogenic strepto B agar (Biolife, Milan, Italy) and incubated at 37 °C in 5% CO_2_. Identification was confirmed by the Dryspot Streptococcal Grouping Kit (Oxoid SpA; Rodano, Milan, Italy) and/or by mass spectrometry MALDI TOF analysis (Bruker Daltonics, Bremen, Germany) [52].

Antimicrobial susceptibility testing was performed by automated broth microdilution method with a Phoenix M50 instrument (Becton Dickinson, Franklin Lakes, NJ, USA) using panel SMIC-ID-11 and/or by E-test gradient strip and qualitative disk diffusion methods. Procedures and results interpretation was carried out according to the EUCAST guidelines (http://www.eucast.org). By automated Phoenix M50 system, the following antimicrobial agents were tested: benzylpenicillin (PEN G), vancomycin (VAN), teicoplanin (TEC), levofloxacin (LVX), linezolid (LZD), chloramphenicol (CHL), clindamycin (CLI), erythromycin (ERY), tetracycline (TET), and high-level gentamicin resistance (HLGR) using the recently proposed clinical cut-off [20].

Macrolide resistance phenotypes were classified as expressing the M phenotype when they were resistant to macrolides only, or to the MLS_B_ phenotype when showing cross-resistance to macrolides and lincosamides, either constitutive (cMLS_B_) or inducible (iMLS_B_) [51,52,53].

PCR screening of the HLGR bifunctional enzyme *aac*(6′)-*aph*(2″) gene was performed using primers and conditions already described [26].

HLGR GBS isolates were studied for the presence of the macrolide resistance genes *erm*(A), *erm*(B), and *mef*, and tetracycline resistance genes *tet*(M) and *tet*(O) by PCR, as already reported [51,52].

Serotyping was performed by using the latex agglutination test ImmuLexTM StrepB-Kit (SSI Diagnostica, HillerØd, Denmark) according to the manufacturer’s instructions [54,55]. Molecular typing by multiplex PCR assay was used both in case of non-typeable strains and for confirming the results of the agglutination test [56].

The presence of GBS alpha (*bca*) and alpha-like (*epsilon*, *rib*, *alp2/3*, *alp4*) surface protein genes was investigated by a multiplex PCR assay [57,58]. PCR screening for pili islands was performed by PCR according to [43].

Genetic element harboring the *aac*(6′)-*aph*(2″) gene was investigated by PCR walking using primers previously described [25,26,29] (Table 4) and following the established process described in [39]. Bakta v1.5 was used to annotate putative MGEs (https://github.com/oschwengers/bakta, accessed on 14 January 2024). Pairwise BLASTn alignment of identified MGEs was performed and visualized using Easyfig v2.2.2 [59].

Identification of hypervirulent ST-17 lineage was performed using a PCR assay based on the detection of the *hvgA* gene [60].

Sequence types (STs) and clonal complexes (CCs) of HLGR GBS isolates were obtained by MLST protocol as previously described [50].

WGS of serotype IV ST1010 GBS strains was performed by the Nanopore MinIon technology and high-quality reads were de novo assembled. [49,61]

## 5. Conclusions

The prevalence of serotype IV GBS among adults is increasing in Italy because of an HLGR clonal lineage (ST1010, CC452).

The truncated Tn3706 element carrying the HLGR tract in this clonal lineage is integrated in one side of an ICE already identified in the serotype Ia GBS 515 strain, leading to the new 45 kb long ICESag9931.

The increase in HLGR in GBS could influence the effectiveness of antibiotic therapy in GBS sepsis and meningitis. Therefore, HLGR testing should be included in routine clinical microbiology for GBS.

## Figures and Tables

**Table 1:** ID strain, strain type, year of isolation, serotype, antimicrobial susceptibility testing results, macrolide and tetracycline resistance genotype and phenotype of 20 HLGR-GBS ^a^ isolates included in this study.

ID	Strain Type	Year	st	CLI	ERY	TET	CHL	LZD	LVX	PEN G	VAN	TEC	Macrolide R Genotype/Phenotype	TET R Genotype
				MIC (mg/L), Category (S, I, R)										
9543	A-invD	2016	Ib	>0.5, R	>0.5, R	>4, R	≤2, S	1, S	1, I	0.06, S	≤0.5, S	≤1, S	*erm*(B)/cMLS_B_	*tet*(O)
9646	N-invD	2016	III	0.06, S	≤0.06, S	>4, R	≤2, S	1, S	≤0.5, I	≤0.03, S	≤0.5, S	≤1, S	-	*tet*(M)
9716	N-invD	2016	III	0.125, S	≤0.06, S	>4, R	≤2, S	1, S	1, I	≤0.03, S	≤0.5, S	≤1, S	-	*tet*(M)
9834	A-invD	2018	IV	0.06, S	≤0.06, S	≤0.5, S	≤2, S	1, S	≤0.5, I	≤0.03, S	≤0.5, S	≤1, S	-	-
9879	A-invD	2019	IV	0.06, S	≤0.06, S	≤0.5, S	≤2, S	1, S	≤0.5, I	0.06, S	≤0.5, S	≤1, S	-	-
9907	A-invD	2019	IV	0.06, S	≤0.06, S	≤0.5, S	≤2, S	1, S	≤0.5, I	0.06, S	≤0.5, S	≤1, S	-	-
9912	A-invD	2019	IV	0.06, S	≤0.06, S	≤0.5, S	≤2, S	1, S	≤0.5, I	0.06, S	≤0.5, S	≤1, S	-	-
9931	N-invD	2021	IV	0.06, S	≤0.06, S	≤0.5, S	≤2, S	1, S	≤0.5, I	0.06, S	≤0.5, S	≤1, S	-	-
10027	A-carr	2020	IV	0.06, S	≤0.06, S	≤0.5, S	≤2, S	1, S	≤0.5, I	0.06, S	≤0.5, S	≤1, S	-	-
10042	A-carr	2020	IV	0.06, S	≤0.06, S	≤0.5, S	≤2, S	1, S	≤0.5, I	0.06, S	≤0.5, S	≤1, S	-	-
10051	A-carr	2020	IV	0.06, S	≤0.06, S	≤0.5, S	≤2, S	1, S	≤0.5, I	0.06, S	≤0.5, S	≤1, S	-	-
10066	A-carr	2020	IV	0.06, S	≤0.06, S	≤0.5, S	≤2, S	1, S	≤0.5, I	0.06, S	≤0.5, S	≤1, S	-	-
10220	A-carr	2020	IV	0.06, S	≤0.06, S	≤0.5, S	≤2, S	1, S	≤0.5, I	0.06, S	≤0.5, S	≤1, S	-	-
10239	A-carr	2020	IV	0.06, S	≤0.06, S	≤0.5, S	≤2, S	1, S	≤0.5, I	0.06, S	≤0.5, S	≤1, S	-	-
10251	A-carr	2020	IV	0.06, S	≤0.06, S	≤0.5, S	≤2, S	1, S	≤0.5, I	0.06, S	≤0.5, S	≤1, S	-	-
10272	A-carr	2020	IV	0.06, S	≤0.06, S	≤0.5, S	≤2, S	1, S	≤0.5, I	0.06, S	≤0.5, S	≤1, S	-	-
10273	A-carr	2020	IV	0.06, S	≤0.06, S	≤0.5, S	≤2, S	1, S	≤0.5, I	0.06, S	≤0.5, S	≤1, S	-	-
9714	A-inv	2016	V	0.06, S	0.125, S	>4, R	≤2, S	1, S	>4, R	≤0.03, S	≤0.5, S	≤1, S	-	*tet*(M)
9976	A-carr	2020	V	>0.5, R	>0.5, R	>4, R	≤2, S	1, S	>4, R	≤0.03, S	≤0.5, S	≤1, S	*erm*(A)/iMLS_B_	*tet*(M)
10039	A-carr	2020	V	0.06, S	>0.5, R	>4, R	≤2, S	1, S	>4, R	≤0.03, S	≤0.5, S	≤1, S	*erm*(A)/iMLS_B_	*tet*(M)

A-invD: adult invasive disease; N-invD: neonatal invasive disease; A-carr: pregnant women carriage; st: serotype; CLI: clindamycin; ERY: erythromycin; TET: tetracycline; CHL: chloramphenicol; LZD: linezolid; LVX: levofloxacin; PEN G: penicillin G; VAN: vancomycin; TEC: teicoplanin. S: susceptible standard dose; I: susceptible, increased dose; R: resistant (EUCAST recommendations). ^a^ Clinical isolates of GBS tested for susceptibility to gentamicin are considered HLGR-GBS with a gentamicin MIC value ≥ 512 mg/L, or diameter size < 8 mm using 120 µg disk of gentamicin [20].

**Table 2:** Serotype, source and year of isolation, surface Alp proteins, *hvgA*, pili islands (PI), sequence type (ST), and Clonal Complex (CC) of the 20 HLGR-GBS isolates.

Serotype	Year (n)	Surface Proteins Genes (n)	*hvgA* Gene (n)	PI (n)	ST (n)	CC
IV	2018 (1 A-invD); 2019 (3 A-invD); 2020 (9 A-carr); 2021 (N-invD)	*alpha-C* (14)	Positive (14)	PI-2b (14)	1010 (14)	452
V	2016 (1 A-invD), 2020 (2 A-carr)	*alp1* (3)	Negative (3)	PI-1 + 2a (3)	19 (3)	19
III	2016 (2 N-invD)	*rib* (2)	Positive (2)	PI-1 + 2b (2)	17 (2)	17
Ib	2016 (1 A-invD)	*alpha-C*	Negative	PI-2a	12	12

**Table 3:** PCR mapping of the transposons carrying the gentamicin resistance gene.

Serotype (n)	Transposon Structure			
	*aac*(6′)-*aph*(2″) Gene	IS256L	3′-inter	5′-inter
Ib (1)	+	+	+	+
III (2)	+	+	−	+
IV (14)	+	+	−	+
V (3)	+	+	−	+

Transposon structure was determined by PCR mapping according to Zhang et al., 2018 [25]): ‘+’ represents positive PCR result and ‘−’ represents negative PCR result. The primers and amplicon sizes (bp) are listed in Table 4. IS256L: covering most of the *IS256* gene; 3′-inter: intergenic region between *IS256* and the 3′ portion of *aac*(6′)-*aph*(2″) gene; 5′-inter: intergenic region between *IS256* and the 5’ portion of *aac*(6’)-*aph*(2”) gene.

**Table 4:** Primers used in this study to determine the structure of the transposon carrying the *aac*(6′)-*aph*(2″) gene in our HLGR-GBS isolates.

Fragment Name	Primers	Amplicon Size (bp)
*aac*(6′)-*aph*(2″)	F1: 5′-CAGAGCCTTGGGAAGATGAAG-3′	348 (from Vakulenko et al. AAC 2003; 47:1423) [26]
	R1: 5′-CCTCGTGTAATTCATGTTCTGGC-3′	
3′-inter	F3: 5′-GATATATTAAGAATGTATGG-3′	371 (from Zhang et al. IJAA 2018; 52:799) [25]
	R3: 5′-GAGCCGTTCTTATGGACCTAC-3’	
5′-inter	F2: 5′-GAGCCGTTCTTATGGACCTAC-3’	628 (from Zhang et al. IJAA 2018; 52:799) [25]
	R2: 5′-CCACCATAAAATTCTAATAC-3’	
IS256L	F5: 5′-TGAAAAGCGAAGAGATTCAAA GC-3′	1103 (from Zhang et al. IJAA 2018; 52:799) [25]
	R5: 5′-ATGTAGGTCCATAAGAACGGC-3′	

IS256L: covering most of the *IS256* gene; 3′-inter: intergenic region between *IS256* and the 3′ portion of *aac*(6′)-*aph*(2″) gene; 5′-inter: intergenic region between *IS256* and the 5′ portion of *aac*(6′)-*aph*(2″) gene.

## Data Availability

The original contributions presented in the study are included in the article. Further inquiries can be directed to the corresponding author.

## References

[1] Madrid L., Seale A.C., Kohli-Lynch M., Edmond K.M., Lawn J.E., Heath P.T., Madhi S.A., Baker C.J., Bartlett L., Cutland C. (2017). Infant GBS Disease Investigator Group. Infant group B streptococcal disease incidence and serotypes worldwide: Systematic review and meta-analyses. Clin. Infect. Dis..

[2] Furfaro L.L., Chang B.J., Payne M.S. (2018). Perinatal *Streptococcus agalactiae* epidemiology and surveillance targets. Clin. Microbiol. Rev..

[3] Schrag S.J., Verani J.R. (2013). Intrapartum antibiotic prophylaxis for the prevention of perinatal group B streptococcal disease: Experience in the United States and implications for a potential group B streptococcal vaccine. Vaccine.

[4] Francois Watkins L.K., McGee L., Schrag S.J., Beall B., Jain J.H., Pondo T., Farley M.M., Harrison L.H., Zansky S.M., Baumbach J. (2019). Epidemiology of invasive group B streptococcal infections among nonpregnant adults in the United States, 2008–2016. JAMA Intern. Med..

[5] Cieslewicz M.J., Chaffin D., Glusman G., Kasper D., Madan A., Rodrigues S., Fahey J., Wessels M.R., Rubens C.E. (2005). Structural and genetic diversity of group B streptococcus capsular polysaccharides. Infect. Immun..

[6] Ferrieri P., Lynfield R., Creti R., Flores A.E. (2013). Serotype IV and invasive group B *Streptococcus* disease in neonates, Minnesota, USA, 2000–2010. Emerg. Infect. Dis..

[7] Nanduri S.A., Petit S., Smelser C., Apostol M., Alden N.B., Harrison L.H., Lynfield R., Vagnone P.S., Burzlaff K., Spina N.L. (2019). Epidemiology of invasive early-onset and late-onset group B streptococcal disease in the United States, 2006 to 2015: Multistate laboratory and population-based surveillance. JAMA Pediatr..

[8] Kimura K., Suzuki S., Wachino J., Kurokawa H., Yamane K., Shibata N., Nagano N., Kato H., Shibayama K., Arakawa Y. (2008). First molecular characterization of group B streptococci with reduced penicillin susceptibility. Antimicrob. Agents Chemother..

[9] Dahesh S., Hensler M.E., Van Sorge N.M., Gertz R.E., Schrag S., Nizet V., Beall B.W. (2008). Point mutation in the group B streptococcal *pbp2x* gene conferring decreased susceptibility to beta-lactam antibiotics. Antimicrob. Agents Chemother..

[10] Kimura K., Nagano N., Arakawa Y. (2015). Classification of group B streptococci with reduced β-lactam susceptibility (GBS-RBS) based on the amino acid substitutions in PBPs. J. Antimicrob. Chemother..

[11] Metcalf B.J., Chochua S., Gertz R.E., Hawkins P.A., Ricaldi J., Li Z., Walker H., Tran T., Rivers J., Mathis S. (2017). Short-read whole genome sequencing for determination of antimicrobial resistance mechanisms and capsular serotypes of current invasive *Streptococcus agalactiae* recovered in the USA. Clin. Microbiol. Infect..

[12] Koide S., Nagano Y., Takizawa S., Sakaguchi K., Soga E., Hayashi W., Tanabe M., Denda T., Kimura K., Arakawa Y. (2022). Genomic traits associated with virulence and antimicrobial resistance of invasive group B *Streptococcus* isolates with reduced penicillin susceptibility from elderly adults. Microbiol. Spectr..

[13] American College of Obstetricians and Gynecologists Prevention of Early-Onset Group B *Streptococcal* Disease in Newborns. 2019. No. 782. https://www.acog.org/clinical/clinical-guidance/committee-opinion/articles/2020/02/prevention-of-group-b-streptococcal-early-onset-disease-in-newborns?utm_source=vanity&utm_medium=web&utm_campaign=clinical.

[14] Sendi P., Furitsch M., Mauerer S., Florindo C., Kahl B.C., Shabayek S., Berner R., Spellerberg B. (2016). Chromosomally and extrachromosomally mediated high-level gentamicin resistance in *Streptococcus agalactiae*. Antimicrob. Agents Chemother..

[15] Vekemans J., Crofts J., Baker C.J., Goldblatt D., Heath P.T., Madhi S.A., Le Doare K., Andrews N., Pollard A.J., Saha S.K. (2019). The role of immune correlates of protection on the pathway to licensure, policy decision and use of group B *Streptococcus* vaccines for maternal immunization: Considerations from World Health Organization consultations. Vaccine.

[16] Buurman E.T., Timofeyeva Y., Gu J., Kim J.H., Kodali S., Liu Y., Mininni T., Moghazeh S., Pavliakova D., Singer C. (2019). A novel hexavalent capsular polysaccharide conjugate vaccine (GBS6) for the prevention of neonatal group B streptococcal infections by maternal immunization. J. Infect. Dis..

[17] Paoletti L.C., Kasper D.L. (2019). Surface structures of group B *Streptococcus* important in human immunity. Microbiol. Spectr..

[18] Bradley J.S., Barnett E.D., Cantey J.B. (2021). Nelson’s Pediatric Antimicrobial Therapy 2021.

[19] Clinical and Laboratory Standards Institute (2020). Performance Standards for Antimicrobial Susceptibility Testing.

[20] Creti R., Imperi M., Berardi A., Angeletti S., Gherardi G. (2022). Laboratory breakpoints for assessing high level gentamicin resistance in *Streptococcus agalactiae*: It is the time for a consensus. Clin. Microbiol. Infect..

[21] Carreras-Abad C., Ramkhelawon L., Heath P.T., Le Doare K. (2020). A vaccine against group B *Streptococcus*: Recent advances. Infect. Drug Resist..

[22] McGee L., Chochua S., Li Z., Mathis S., Rivers J., Metcalf B., Ryan A., Alden N., Farley M.M., Harrison L.H. (2021). Multistate, population-based distributions of candidate vaccine targets, clonal complexes, and resistance features of invasive group B streptococci within the United States, 2015–2017. Clin. Infect. Dis..

[23] Tazi A., Disson O., Bellais S., Bouaboud A., Dmytruk N., Dramsi S., Mistou M.Y., Khun H., Mechler C., Tardieux I. (2010). The surface protein HvgA mediates group B streptococcus hypervirulence and meningeal tropism in neonates. J. Exp. Med..

[24] Schindler Y., Rahav G., Nissan I., Treygerman O., Prajgrod G., Attia B.Z., Raz R., Valenci G.Z., Tekes-Manova D., Maor Y. (2023). Group B streptococcus virulence factors associated with different clinical syndromes: Asymptomatic carriage in pregnant women and early-onset disease in the newborn. Front. Microbiol..

[25] Zhang J.M., Wang Q., Han T.Y., Liu J.H., Hu X.X., Qiao F., Yang X.Y., Li C.R., You X.F. (2018). Structure analysis of transposons carrying the *aac(6’)-aph(2″)* gene in *Enterococcus faecalis* isolated in Beijing, China, and comparison of their transfer efficiency. Int. J. Antimicrob. Agents..

[26] Vakulenko S.B., Donabedian S.M., Voskresenskiy A.M., Zervos M.J., Lerner S.A., Chow J.W. (2003). Multiplex PCR for detection of aminoglycoside resistance genes in enterococci. Antimicrob. Agents Chemother..

[27] Hodel-Christian S.L., Murray B.E. (1991). Characterization of the gentamicin resistance transposon Tn5281 from *Enterococcus faecalis* and comparison to the staphylococcal transposons, Tn4001 and Tn4031. Antimicrob. Agents Chemother..

[28] Lyon B.R., May J.W., Skuray R.A. (1984). Tn4001: A gentamicin and kanamycin resistance transposon in *Staphylococcus aureus*. Mol. Gen. Genet..

[29] Horaud T., de Céspèdes G., Trieu-Cuot P. (1996). Chromosomal gentamicin resistance transposon *Tn3706* in *Streptococcus agalactiae* B128. Antimicrob. Agents Chemother..

[30] Nagaoka K., Konno S., Murase K., Kikuchi T., Nakagawa I. (2018). Complete Genome Sequence of *Streptococcus agalactiae* Serotype III, Multilocus Sequence Type 335 Strain HU-GS5823, Isolated from a Human Patient in Japan with Severe Invasive Infection. Microbiol. Resour. Announc..

[31] Chuzeville S., Puymège A., Madec J.Y., Haenni M., Payot S. (2012). Characterization of a new CAMP factor carried by an integrative and conjugative element in *Streptococcus agalactiae* and spreading in Streptococci. PLoS ONE.

[32] Ku T.S.N., Al Mohajer M., Newton J.A., Wilson M.H., Monsees E., Hayden M.K., Messacar K., Kisgen J.J., Diekema D.J., Morgan D.J. (2023). Improving antimicrobial use through better diagnosis: The relationship between diagnostic stewardship and antimicrobial stewardship. Infect. Control Hosp. Epidemiol..

[33] Butler M.S., Gigante V., Sati H., Paulin S., Al-Sulaiman L., Rex J.H., Fernandes P., Arias C.A., Paul M., Thwaites G.E. (2022). Analysis of the Clinical Pipeline of Treatments for Drug-Resistant Bacterial Infections: Despite Progress, More Action Is Needed. Antimicrob. Agents Chemother..

[34] Doumith M., Mushtaq S., Martin V., Chaudhry A., Adkin R., Coelho J., Chalker V., MacGowan A., Woodford N., Livermore D.M. (2017). Genomic sequences of *Streptococcus agalactiae* with high-level gentamicin resistance, collected in the BSAC bacteraemia surveillance. J. Antimicrob. Chemother..

[35] Buu-Hoï A., Le Bouguenec C., Horaud T. (1990). High-level chromosomal gentamicin resistance in *Streptococcus agalactiae* (group B). Antimicrob. Agents Chemother..

[36] Liddy H., Holliman R. (2002). Group B Streptococcus highly resistant to gentamicin. J. Antimicrob. Chemother..

[37] Zhi Y., Ji H.J., Jung J.H., Byun E.B., Kim W.S., Lin S.M., Lim S., Jang A.Y., Choi M.J., Ahn K.B. (2021). Molecular characteristics of IS1216 carrying multidrug resistance gene cluster in serotype III/sequence type 19 group B streptococcus. mSphere.

[38] Khan U.B., Portal E.A.R., Sands K., Lo S., Chalker V.J., Jauneikaite E., Spiller O.B. (2023). Genomic Analysis Reveals New Integrative Conjugal Elements and Transposons in GBS Conferring Antimicrobial Resistance. Antibiotics.

[39] Puymège A., Bertin S., Guédon G., Payot S. (2015). Analysis of *Streptococcus agalactiae* pan-genome for prevalence, diversity and functionality of integrative and conjugative or mobilizable elements integrated in the tRNA(Lys CTT) gene. Mol. Genet. Genom..

[40] Ruppen C., Hemphill A., Sendi P. (2017). In vitro activity of gentamicin as an adjunct to penicillin against biofilm group B Streptococcus. J. Antimicrob. Chemother..

[41] Campisi E., Rinaudo C.D., Donati C., Barucco M., Torricelli G., Edwards M.S., Baker C.J., Margarit I., Rosini R. (2016). Serotype IV *Streptococcus agalactiae* ST-452 has arisen from large genomic recombination events between CC23 and the hypervirulent CC17 lineages. Sci. Rep..

[42] Springman A.C., Lacher D.W., Waymire E.A., Wengert S.L., Singh P., Zadoks R.N., Davies H.D., Manning S.D. (2014). Pilus distribution among lineages of group B *Streptococcus*: An evolutionary and clinical perspective. BMC Microbiol..

[43] Parker R., Laut C., Gaddy J.A., Zadoks R.N., Davies H.D., Manning S.D. (2016). Association between genotypic diversity and biofilm production in group B Streptococcus. BMC Microbiol..

[44] Lacasse M., Valentin A.S., Corvec S., Bémer P., Jolivet-Gougeon A., Plouzeau C., Tandé D., Mereghetti L., Bernard L., Lartigue M.F. (2022). Genotypic Characterization and biofilm production of group B *Streptococcus* strains isolated from bone and joint infections. Microbiol. Spectr..

[45] Jin Z., Li J., Zhou H., Wang Z., Yi L., Liu N., Du J., Chang C.Y., Ji W. (2022). Serotype distribution, virulence determinants and antimicrobial susceptibility of *Streptococcus agalactiae* isolated from young infants. Pathogens.

[46] Zhang L., Ma L., Zhu L., Zhou X.H., Xu L.J., Guo C., Meng J.H., Zhang X.H., Liu Q.H., Huang R. (2021). Molecular characterization of pathogenic group B streptococcus from a tertiary hospital in Shanxi, China: High incidence of sequence type 10 strains in infants/pregnant women. J. Microbiol. Immunol. Infect..

[47] Nabavinia M., Khalili M.B., Sadeh M., Eslami G., Vakili M., Azartoos N., Mojibiyan M. (2020). Distribution of Pilus island and antibiotic resistance genes in *Streptococcus agalactiae* obtained from vagina of pregnant women in Yazd, Iran. Iran. J. Microbiol..

[48] Graux E., Hites M., Martiny D., Maillart E., Delforge M., Melin P., Dauby N. (2021). Invasive group B *Streptococcus* among non-pregnant adults in Brussels-Capital Region, 2005–2019. Eur. J. Clin. Microbiol. Infect. Dis..

[49] Gherardi G., Imperi M., Baldassarri L., Pataracchia M., Alfarone G., Recchia S., Orefici G., Dicuonzo G., Creti R. (2007). Molecular epidemiology and distribution of serotypes, surface proteins, and antibiotic resistance among group B streptococci in Italy. J. Clin. Microbiol..

[50] Creti R., Imperi M., Berardi A., Pataracchia M., Recchia S., Alfarone G., Baldassarri L., Italian Neonatal GBS Infections Working Group (2017). Neonatal group B *Streptococcus* infections: Prevention strategies, clinical and microbiologic characteristics in 7 years of surveillance. Pediatr. Infect. Dis. J..

[51] Creti R., Imperi M., Berardi A., Lindh E., Alfarone G., Pataracchia M., Recchia S. (2021). The Italian Network on Neonatal And Infant Gbs Infections. Invasive Group B Streptococcal Disease in Neonates and Infants, Italy, Years 2015–2019. Microorganisms.

[52] Imperi M., Gherardi G., Berardi A., Baldassarri L., Pataracchia M., Dicuonzo G., Orefici G., Creti R. (2011). Invasive neonatal GBS infections from an area-based surveillance study in Italy. Clin. Microbiol. Infect..

[53] Giovanetti E., Montanari M.P., Mingoia M., Varaldo P.E. (1999). Phenotypes and genotypes of erythromycin-resistant *Streptococcus pyogenes* strains in Italy and heterogeneity of inducibly resistant strains. Antimicrob. Agents Chemother..

[54] Afshar B., Broughton K., Creti R., Decheva A., Hufnagel M., Kriz P., Lambertsen L., Lovgren M., Melin P., Orefici G. (2011). International external quality assurance for laboratory identification and typing of *Streptococcus agalactiae* (Group B streptococci) *J*. Clin. Microbiol..

[55] Slotved H.C., Hoffmann S. (2017). Evaluation of procedures for typing of group B *Streptococcus*: A retrospective study. PeerJ.

[56] Imperi M., Pataracchia M., Alfarone G., Baldassarri L., Orefici G., Creti R. (2010). A multiplex PCR assay for the direct identification of the capsular type (Ia to IX) of *Streptococcus agalactiae*. J. Microbiol. Methods.

[57] Creti R., Fabretti F., Orefici G., von Hunolstein C. (2004). Multiplex PCR assay for direct identification of group B streptococcal alphaprotein-like protein genes. J. Clin. Microbiol..

[58] Martins E.R., Melo-Cristino J., Ramirez M. (2010). Evidence for rare capsular switching in *Streptococcus agalactiae*. J. Bacteriol..

[59] Sullivan M.J., Petty N.K., Beatson S.A. (2011). Easyfig: A genome comparison visualizer. Bioinformatics.

[60] Lamy M.C., Dramsi S., Billoët A., Réglier-Poupet H., Tazi A., Raymond J., Guérin F., Couvé E., Kunst F., Glaser P. (2006). Rapid detection of the “highly virulent” group B *Streptococcus* ST-17 clone. Microbes Infect..

[61] Meehan M., Eogan M., McCallion N., Cunney R., Bray J.E., Jolley K.A., Unitt A., Maiden M.C.J., Harrison O.B., Drew R.J. (2021). Genomic epidemiology of group B streptococci spanning 10 years in an Irish maternity hospital, 2008–2017. J. Infect..

